# Bu-Yin-Qian-Zheng Formula Ameliorates MPP^+^-Induced Mitochondrial Dysfunction in Parkinson’s Disease via Parkin

**DOI:** 10.3389/fphar.2020.577017

**Published:** 2020-12-18

**Authors:** Hao-Jie Ma, Cong Gai, Yuan Chai, Wan-Di Feng, Cui-Cui Cheng, Jin-Kun Zhang, Yu-Xin Zhang, Lu-Ping Yang, Zhen-Yu Guo, Yu-Shan Gao, Hong-Mei Sun

**Affiliations:** ^1^ Department of Anatomy, School of Chinese Medicine, Beijing University of Chinese Medicine, Beijing, China; ^2^ Dongfang Hospital, Beijing University of Chinese Medicine, Beijing, China; ^3^ Center for Scientific Research, School of Chinese Medicine, Beijing University of Chinese Medicine, Beijing, China

**Keywords:** mitochondria, MPP, parkin, Bu-Yin-Qian-Zheng formula, Parkinson’s disease

## Abstract

As a typical traditional Chinese medicine, Bu-Yin-Qian-Zheng Formula (BYQZF) has been shown to have neuroprotective effects in patients with Parkinson’s disease (PD), particularly by ameliorating mitochondrial dysfunction and regulating expression of the parkin protein. However, the underlying mechanisms by which BYQZF affects mitochondrial function through parkin are unclear. Accordingly, in this study, we evaluated the mechanisms by which BYQZF ameliorates mitochondrial dysfunction through parkin in PD. We constructed a parkin-knockdown cell model and performed fluorescence microscopy to observe transfected SH-SY5Y cells. Quantitative real-time reverse transcription polymerase chain reaction and western blotting were conducted to detect the mRNA and protein expression levels of parkin. Additionally, we evaluated the cell survival rates, ATP levels, mitochondrial membrane potential (ΔΨm), mitochondrial morphology, parkin protein expression, PINK1 protein expression, and mitochondrial fusion and fission protein expression after treatment with MPP^+^ and BYQZF. Our results showed that cell survival rates, ATP levels, ΔΨm, mitochondrial morphology, parkin protein levels, PINK1 protein levels, and mitochondrial fusion protein levels were reduced after MPP^+^ treatment. In contrast, mitochondrial fission protein levels were increased after MPP^+^ treatment. Moreover, after transient transfection with a negative control plasmid, the above indices were significantly increased by BYQZF. However, there were no obvious differences in these indices after transient transfection with a parkin-knockdown plasmid. Our findings suggest that BYQZF has protective effects on mitochondrial function in MPP^+^-induced SH-SY5Y cells via parkin-dependent regulation of mitochondrial dynamics.

## Introduction

Parkinson’s disease (PD) is a common progressive neurodegenerative disease manifested by motor and nonmotor symptoms (Kalia and Lang, 2015) with degeneration of dopaminergic neurons in the substantia nigra pars compacta (Song et al., 2010; Ham et al., 2020). PD mainly occurs in people over 60 years of age (Grünewald et al., 2019) and is a major burden on social and economic development, particularly as society ages. Therefore, the pathogenesis of PD has been widely studied (Golpich et al., 2017; Obeso et al., 2017; Chen et al., 2018). Mitochondrial dysfunction has been shown to play a central role in the pathogenesis of PD (Exner et al., 2012). Mitochondria are distributed in a highly dynamic tubular network that is constantly reshaped by fusion and fission. Changes in fusion or fission lead to mitochondrial fragmentation, limiting mitochondrial motility and decreasing ATP production, thereby resulting in mitochondrial dysfunction (Luo et al., 2015; Rao et al., 2020). Additionally, the gene encoding parkin, which is related to mitochondrial function and mitophagy, is a genetic susceptibility gene in patients with PD (Kitada et al., 1998). Mutation or deletion of the gene encoding parkin can trigger accumulation of dysfunctional mitochondria (Bankapalli et al., 2020) and cause the death of dopaminergic neurons in PD (Zhu et al., 2018). Because of the substantial role of mitochondrial dysfunction in PD, mitochondrion-targeted therapeutics may facilitate the development of novel drugs for treating PD (Camilleri and Vassallo, 2014; Park et al., 2018; Grünewald et al., 2019; Bai et al., 2020), and parkin may be an important mitochondrial target for expanding our understanding of the molecular mechanisms of mitochondrial dysfunction in PD therapy.

Currently, symptomatic treatment with drugs that stimulate dopamine receptors is used to treat PD (Kalia and Lang, 2015); however, these drugs have major side effects (Kordower, 2015). To reduce and counteract these effects, traditional Chinese medicine has been studied and applied (Chua et al., 2017; Liu et al., 2020). The traditional Chinese medicine Bu-Yin-Qian-Zheng formula (BYQZF) has been widely used to treat PD and its complications. BYQZF is composed of Da-Bu-Yin-Wan (Great Yin Tonic Pill) and Qian-Zheng-San (Symmetry Leading Powder). Our previous studies showed that Da-Bu-Yin-Wan ameliorates MPTP-induced behavioral impairment, increases the expression of tyrosine hydroxylase in the substantia nigra, and prevents loss of substantia nigra dopaminergic neurons in a PD mouse model (Gong et al., 2014). Moreover, Da-Bu-Yin-Wan and/or Qian-Zheng-San significantly increase ATP levels and complex I activity in the midbrain tissue of PD model mice (Zhang et al., 2013), and BYQZF reduced mitochondrial damage and improved the mitochondrial morphological structure in a cellular model of PD (Zhang et al., 2018; Gai et al., 2019). These studies suggest that BYQZF had neuroprotective effects on mitochondrial function and may be applicable as a mitochondrion-targeted therapeutic drug for treating PD. However, the mechanisms by which BYQZF ameliorates mitochondrial dysfunction via parkin are unclear.

Accordingly, in this study, we evaluated the potential mechanisms by which BYQZF ameliorated mitochondrial dysfunction through parkin in MPP^+^-induced SH-SY5Y cells. Our findings provide important insights into the roles of BYQZF and parkin in MPP^+^-induced SH-SY5Y cells.

## Materials and Methods

### Chemical Teagents and Antibodies

SH-SY5Y human neuroblastoma cells were acquired from Shanghai GeneChem Co., Ltd. 1-Methyl-4-phenylpyridinium (MPP^+^) was obtained from Sigma Corporation (St. Louis, MO, United States), and a Cell Counting Kit-8 (CCK-8) was obtained from Dojindo Molecular Technologies. Lipofectamine 3,000, MitoTracker^®^ Red CMXRos (MTR), and tetramethylrhodamine were obtained from Life Technologies and the ATP detection reagent kit was obtained from Beyotime Biotechnology Co., Ltd. The BCA Protein Assay Kit and enhanced chemiluminescence (ECL) autoradiography kit were obtained from Applygen Technologies, Inc.

Anti-mitofusin (MFN) 1, anti-dynamin-related protein 1 (DRP1), and anti-optic atrophy 1 (OPA1) antibodies were obtained from Abcam (Cambridge, United Kingdom). Anti-parkin, anti-MFN2, anti-fission mitochondrial 1 (FIS1), and anti-β-actin polyclonal antibodies were obtained from Proteintech. All secondary antibodies (1:2000) were obtained from Zhong-Shan Golden Bridge Biotechnology.

### Preparation and Analysis for Bu-Yin-Qian-Zheng Formula

BYQZF was composed of Rehmannia glutinosa (Gaertn.) DC. (Shu-Di-Huang), Phellodendron chinense C.K.Schneid. (Huang-Bai), Anemarrhena asphodeloides Bunge (Zhi-Mu), Plastrum Testudinis (Gui-Ban),Sauromatum giganteum (Engl.) Cusimano & Hett. (Bai-Fu-Zi), Bombyx Batryticatus (Jiang-Can), and Scorpio (Quan-Xie)(Chan et al., 2012). All traditional Chinese medicine herbs were purchased from Tong-Ren-Tang Drugstore (Beijing, China) and authenticated by experts in pharmacognosy, as shown in Table 1. To prepare the decoctions, exact amounts of herbs were weighed according to classic percentages and mixed well. The mixture was soaked in distilled water for 30 min and then boiled in eight volumes of water (v/w) for 1 h; this extraction procedure was carried out twice (Zhang et al., 2013). Finally, the decoction was concentrated to 0.5 g/ml, filtered through a 0.22-μm membrane, divided, and stored as a stock solution at –70°C.

**TABLE 1:** TABLE 1Constituents of BYQZF.

Chinese name	Pharmacopoeia	Common name	Weight (g)	Voucher numbers	
Shu-Di-Huang	Rehmannia glutinosa (Gaertn.) DC.	Prepared rehmannia root	36	L02131669	—
Gui-Ban	Plastrum Testudinis	Tortoise plastron	36	L02131669	—
Zhi-Mu	Anemarrhena asphodeloides Bunge.	Common anemarrhena	24	L02131669	—
Huang-Bai	Phellodendron chinense C.K.Schneid.	Amur cork tree bark	24	L02131669	—
Jiang-Can	*Bombyx* Batryticatus	Stiff silkworm	24	L02131669	—
Bai-Fu-Zi	Sauromatum giganteum (Engl.) Cusimano & Hett.	Giant typhonium tuber	24	L81ZDA00604217	—
Quan-Xie	Scorpio	Chinese scorpion (detoxicated)	24	L02131669	—

BYQZF, bu-yin-qian-zheng formula.

Identification and quantification of the marker compounds in BYQZF were performed according to the method described in the updated Pharmacopeia of People’s Republic of China (Chinese Pharmacopoeia Commission, 2020). Briefly, the marker compounds were analyzed using the ultra-performance liquid chromatography tandem–mass spectrometry (UPLC-MS/MS). The separation was carried out on an Acquity BEH C18 (100 mm × 2.1 mm, 1.7 μm; Waters, Milford, MA, United States) analytical column. The column was maintained at 35°C. Water acidified with acetonitrile as Solvent A and 0.1% of formic acid (v/v) as Solvent B were used for the gradient elution at 0.3 ml/min. The gradient program was: 5–50% A (from 0 to 4.5 min), 50% A (from 4.5 to 6 min), 50–100% A (from 6 to 8 min), 100 to 5% A (from 8 to 8.1 min), 5% A (from 8.1 to 10 min) and injection volume of 2 µl. The experiment was performed on both the positive and negative ion modes. Ion spray voltage: positive ion modes, 5 kV; negative ion modes, −2.8 kV; lens voltage: positive ion modes, 50 V; negative ion modes, −50 V; capillary temperature, 320°C; auxiliary gas temperature, 350°C; sheath gas (N_2_) voltage, 40 arb; auxiliary gas (N_2_) voltage, 10 arb. Full scans were acquired in the range of 200–1,200 m/*z* with a resolution of 70,000 in Full MS and the resolution of 17,500 in dd-MS^2^. Fragmentation amplitude was set at 20, 40, 60 eV.

### Cell Culture, Transfection, and Treatments

SH-SY5Y cells were cultured in Dulbecco’s modified Eagle’s medium supplemented with 10% fetal bovine serum and 1% penicillin/streptomycin in a humidified incubator (37°C, 5% CO_2_). For differentiation experiments, cells were replated into 6-, 12-, or 96-well plates and grown to 70–80% confluence after 24 h.

The human wild-type parkin knockdown plasmid was designed by GeneChem. SH-SY5Y cells were plated at a density of 1 × 10^5^ cells/mL and transfected with parkin-knockdown plasmid using the optimum amount of Lipofectamine 3,000 (Invitrogen, Carlsbad, CA, United States) according to the manufacturer’s protocols. After transfection for 48 h, the expression of green fluorescent protein (GFP) was observed under a fluorescence microscope. Cells were cultured in medium containing MPP^+^ (1 mM) with or without BYQZF (5 μg/ml) for 48 h.

### Experimental Groups

The experiment was divided into seven groups: Control group (C), Negative control group (NC), Negative model group (NM), Negative treatment group (NT), Parkin-knockdown control group (PKC), Parkin-knockdown model group (PKM) and Parkin-knockdown treatment group (PKT). Experimental groups and treatments are shown in Table 2.

**TABLE 2:** Experimental groups and treatments.

Groups	Negative plasmid	Parkin-knockdown plasmid	MPP^+^ (1 mM)	BYQZF (5 μg/ml)
Control (C)	−	−	−	−
Negative control (NC)	+	−	−	−
Negative model (NM)	+	−	+	−
Negative treatment (NT)	+	−	+	+
Parkin-knockdown control (PKC)	−	+	−	−
Parkin-knockdown model (PKM)	−	+	+	−
Parkin-knockdown treatment (PKT)	−	+	+	+

Notes: +, added; −, not added.

### Quantitative Real-Time Reverse Transcription Polymerase Chain Reaction

The mRNA expression of parkin was verified by qRT-PCR using a One-Step SYBR PrimeScript RT-PCR Kit II and quantified on an ABI StepOne Plus sequence detection system under the following conditions: 5 min at 42°C and 10 s at 95.0°C for initial denaturation, followed by 39 cycles of 5 s at 95°C and 20 s at 60°C. The unknown template was calculated using the 2^−ΔΔCT^ method for quantitative analysis. Sequences of the qRT-PCR-specific primers are listed in Table 3.

**TABLE 3:** Sequences of primers used for qRT-PCR.

Name	Sequences
Forward primer for parkin	5′-CAA​GGT​CGG​GCA​GGA​AGA​G-3′
Reverse primer for parkin	5′-TAA​TAC​GAC​TCA​CTA​TAG​GG-3′
Forward primer for *β*-actin	5′-CGG​CTA​CAG​CTT​CAC​CAC​CA-3′
Reverse primer for *β*-actin	5′-CGG​GCA​GCT​CGT​AGC​TCT​TC-3′

qRT-PCR, quantitative real-time reverse transcription polymerase chain reaction.

### Western Blotting

Total protein samples were obtained from SH-SY5Y cells using a BCA Protein Assay kit. First, equal amounts of protein were loaded into the wells and separated by electrophoresis. The proteins were then transferred to polyvinylidene difluoride membranes (Millipore, Billerica, MA, United States), and nonspecific binding was blocked for 1 h with skim milk. The membranes were then incubated with primary antibodies overnight at 4°C followed by incubation at room temperature with horseradish peroxidase-conjugated goat anti-rabbit/mouse IgG (H + L) secondary antibodies. Finally, protein bands were visualized using an ECL autoradiography kit.

### Assessment of Cell Survival Rate

CCK-8 assays were performed to evaluate cell survival rates (Ishiyama et al., 1997). Briefly, 10 μl CCK-8 reagent and 90 μl Dulbecco’s modified Eagle’s medium were added to each well of a 96-well plate. After incubation for 1 h (37°C, 5% CO_2_), the absorbance at 450 nm was measured using a full-wavelength fluorescence microplate (Tecan Safire2; Tecan, Switzerland). The survival rate was calculated as follows:

$$C=\frac{OD_{1}-OD_{2}}{OD_{3}-OD_{2}}\times 100\%$$

where C is the cell survival rate and *OD*_i_ is the optical density (subscripts 1, 2, and 3 represent the experimental group, blank group, and control group, respectively).

### Mitochondrial Membrane Potential (ΔΨm)

Mitochondrial staining was performed using the mitochondrial probe tetramethylrhodamine in living cells to detect the mitochondrial membrane potential followed by observation with an Olympus FV1000 confocal microscope (559 nm excitation/572 nm emission). ImageJ software (Schneider et al., 2012) was used to process the images and measure the mitochondrial fluorescence intensity (OD value) of each image.

### Adenosine Triphosphate Levels

Cellular ATP content was detected using luciferase assays following the manufacturer’s instructions. Briefly, ATP concentrations in the samples were calculated based on a standard curve, and the protein concentration of the sample was detected using a BCA Protein Concentration Assay Kit. Finally, the ATP concentration was converted into nmol/mg protein.

### Confocal Fluorescence Microscopy for Mitochondrial Morphology

Cells were seeded into a 20-mm confocal culture dish. Briefly, the cells were incubated with 100 nM MTR for 15 min at 37°C in the dark before visualization (Pendergrass et al., 2004). Fluorescence was detected (559 nm excitation/603 nm emission) using an Olympus FV1000 confocal microscope. Acquired images were quantified by ImageJ software. The form factor was calculated using the following equation: form factor = *P* ^2^/4π*A* (*P*, perimeter; *A*, area), as previously described (Mortiboys et al., 2008). Mitochondrial fluorescence intensity (OD value) and the mean length and number of mitochondrial network branches were also measured using ImageJ software.

### Statistical Analysis

The data are presented as the means ± standard deviations and were analyzed by Two-Two Comparisons in one-way analysis of variance (One-way ANOVA). LSD(L) and S-N-K(S) were performed to determine *p* values using SPSS20.0 software. Results with *p* values of less than 0.05 between two groups were considered as statistically significant.

## Results

### Analysis of Marker Compounds of Bu-Yin-Qian-Zheng Formula

**FIGURE 1:**
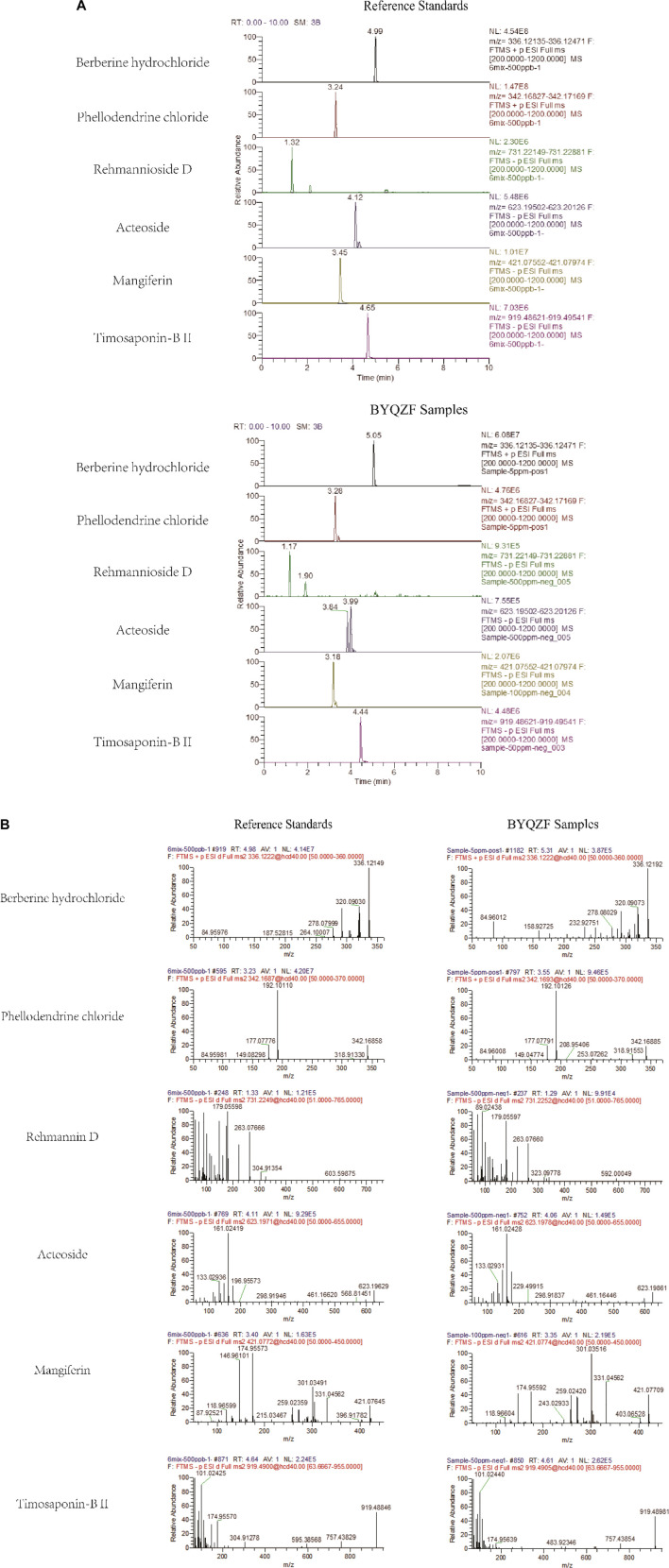
UPLC-MS/MS analysis of BYQZF. Six reference standards were used to identify the marker compounds for BYQZF. **(A)** Chromatograms of BYQZF. **(B)** Secondary debris ion of BYQZF.

The marker compounds in BYQZF were analyzed with UPLC-MS/MS. Chromatograms of the BYQZF analyzed with relative reference standards are shown in Figure 1. Table 4 shows the equations of the calibration curves and limits of quantification of those components determined. All calibration curves showed good linear regression (*r* ^2^ > 0.99). Table 5 shows the results of precision and accuracy tests. Calculated by the calibration curves using an internal standard, it is clearly shown that the concentrations of the six compounds calculated by the calibration curves.

**TABLE 4:** Linear range, *r* ^2^ and limits of quantification of calibration curve used in the determination of the six analytes.

Analyte	Linear range (mg/L)	Calibrtion curve	*r* ^2^
Berberine hydrochloride	152946479–2887233808	y = 2880647076.02 x + 39942604.07	0.9986
Phellodendrine chloride	42732718–785420745	y = 781954206.57 x + 9288819.97	0.9994
Rehmannioside D	1321749–22458970	y = 11064305.68 x + 362681.68	0.9998
Acteoside	1218672–38470222	y = 38505650.16 x + 274315.44	0.9982
Mangiferin	3195648–71815613	y = 71539500.94 x + 1274365.85	0.9976
Timosaponin-B II	1776821–50965360	y = 51020933.82 x + 214533.49	0.9988

**TABLE 5:** The concentrations of the six compounds.

Analyte	Concentration (mg/L)	Peak area
Berberine hydrochloride	0.081	270665163
Phellodendrine chloride	0.1131	97729078
Rehmannioside D	0.2826	3489314
Acteoside	0.0563	2443068
Mangiferin	0.1394	11249558
Timosaponin-B II	0.5883	30230081

### Expression of Green Fluorescent Protein in Transfected Cells

SH-SY5Y cells were transfected with the negative control plasmid and parkin-knockdown plasmid, both of which were labeled with GFP. The expression of GFP was observed by fluorescence microscopy at 48 h after transfection.

**FIGURE 2:**
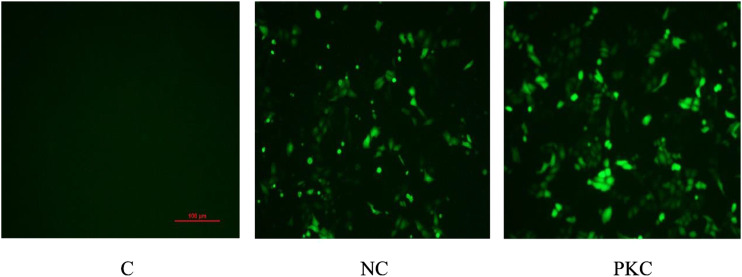
Expression of GFP in SH-SY5Y cells. GFP expression was observed by fluorescence microscopy at 48 h after transfection in SH-SY5Y cells. PKC, parkin-knockdown control group; NC, negative control group.

As shown in Figure 2, the number of cells was high, and the cells showed a plump morphology in both the control and negative control groups. In contrast, in the parkin-knockdown group, the number of cells was reduced, and the cells showed a generally normal morphology. Increased GFP expression was observed in the negative control group and parkin-knockdown control group (Figure 2), suggesting that the plasmid was successfully transfected into the cells.

### Analysis of mRNA and Protein Expression Levels of Parkin

**FIGURE 3:**
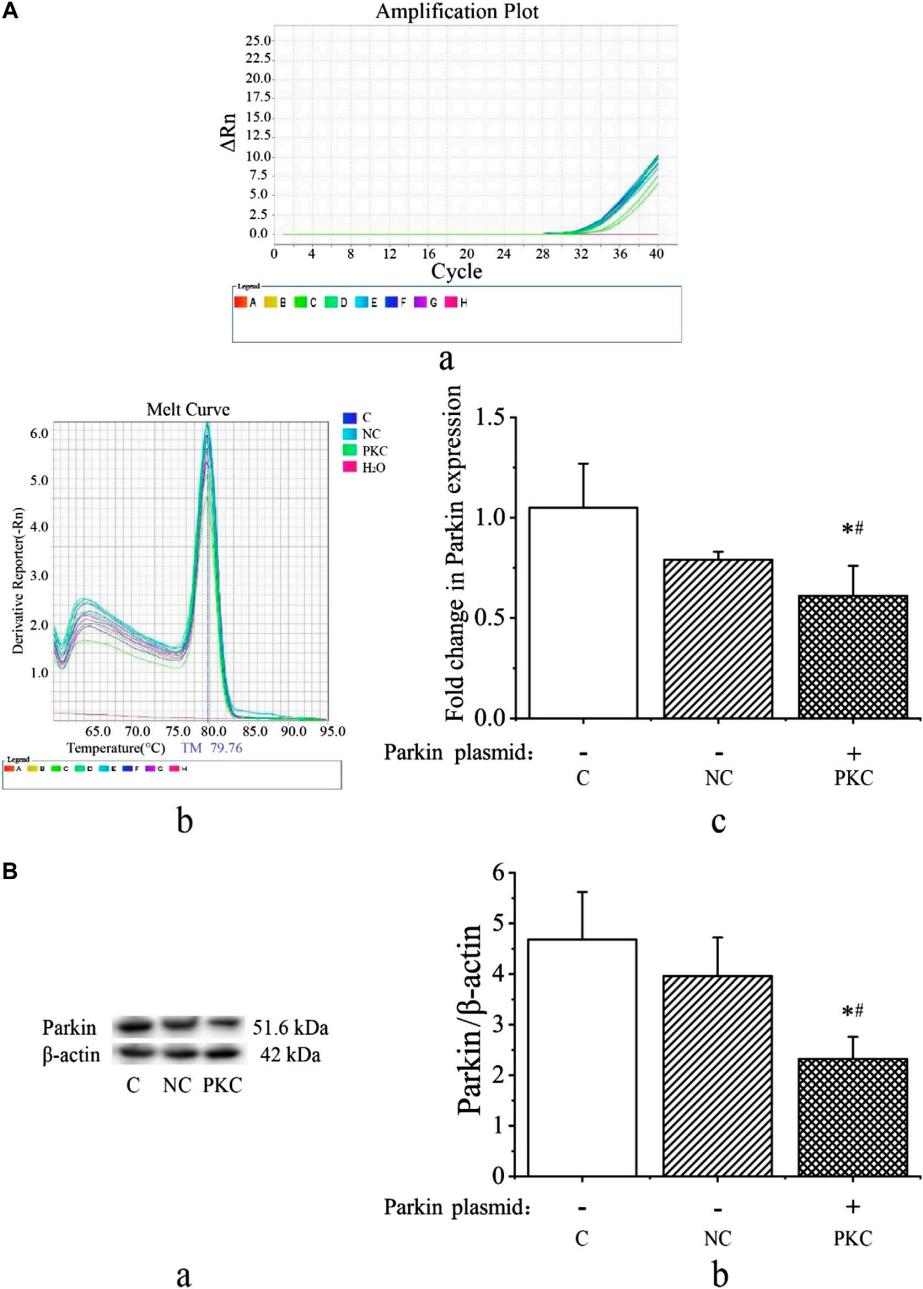
mRNA and protein expressions of parkin in SH-SY5Y cells. Parkin expression was evaluated at 48 h after transfection in SH-SY5Y cells. **(A)** mRNA expression of parkin after transient transfection with a parkin-knockdown plasmid. **(A)** Amplification cycles. **(B)** Melt curve analysis. **(C)** Fold-change in parkin mRNA expression, as quantified using the 2^−ΔΔCT^ method. **(B)** Protein expression of parkin after transient transfection with parkin-knockdown plasmid. **(A)** Western blotting bands. **(B)** Quantification of parkin expression; the bar graph shows the data in arbitrary units (AU) from three independent experiments after normalization to *β*-actin expression. Data are expressed as means ± standard deviations (*n* = 3). Compared with the control **(C)** group, ^∗^ *p* < 0.05; compared with the negative control (NC) group, ^#^ *p* < 0.05.

Next, we investigated parkin expression at 48 h after transfection by qRT-PCR and western blotting. As shown in Figures 3Ac, parkin mRNA expression was decreased in the parkin-knockdown control group (*p* < 0.05). There were no differences between the control and negative control groups (*p* > 0.05). Additionally, melting curve analysis revealed that no primer-dimers were formed during the 40 real-time PCR amplification cycles (Figures 3Aa,b).

As shown in Figure 3B, the expression of parkin protein was visibly decreased in the parkin-knockdown control group (*p* < 0.05). There were no differences between the control and negative control groups (*p* > 0.05). These findings indicate that the parkin-knockdown SH-SY5Y cell line was successfully constructed.

### Effects of Bu-Yin-Qian-Zheng Formula on Cell Survival Rates, Adenosine Triphosphate Levels, and ΔΨm in Parkin-Knockdown Parkinson’s Disease Cells

**FIGURE 4:**
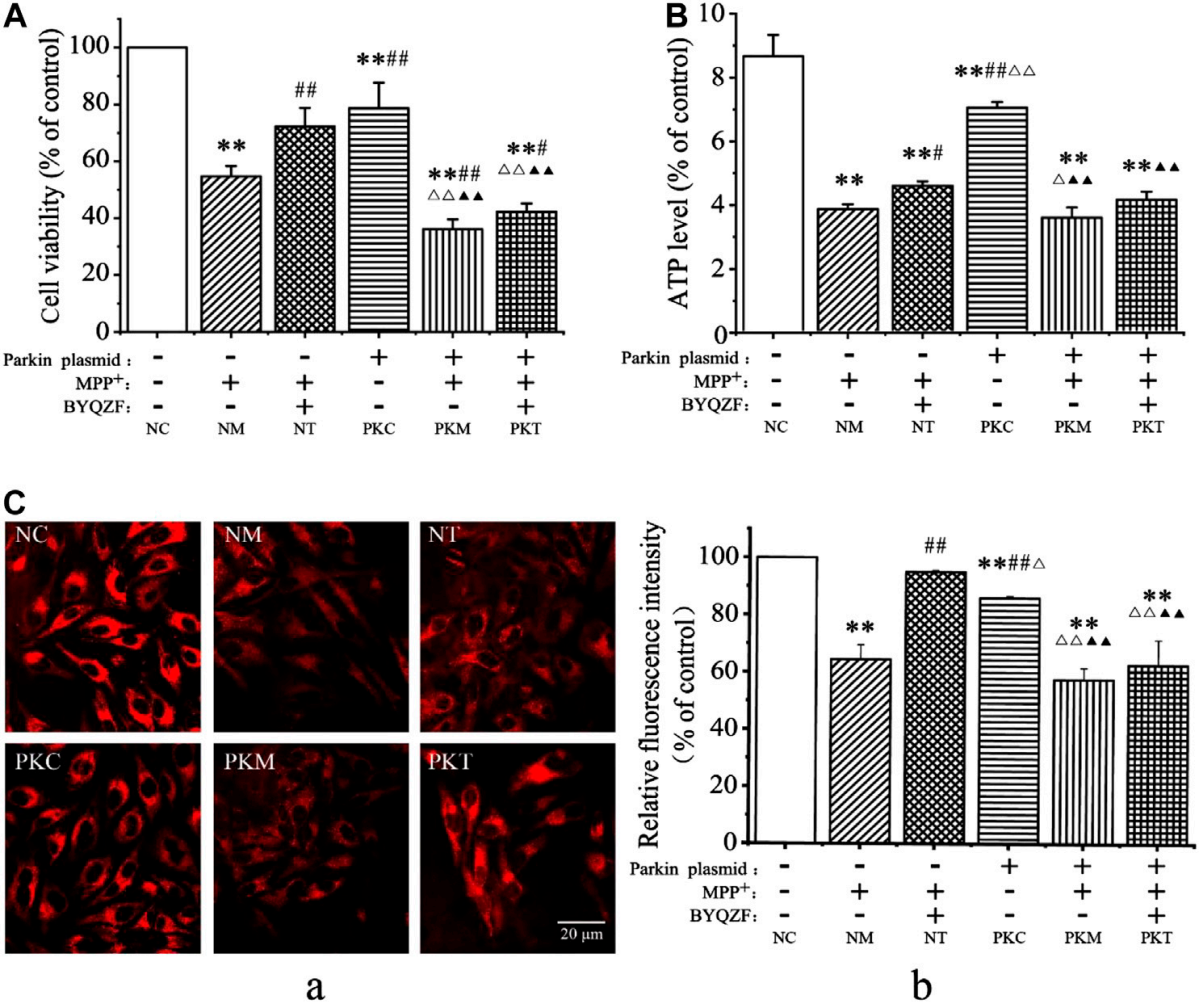
Analysis of cell survival rates and mitochondrial function. **(A)** Effects of BYQZF on the survival rate of PD cells after transfection with a parkin-knockdown plasmid. **(B)** Effects of BYQZF on ATP levels in PD cells after transfection with a parkin-knockdown plasmid. **(C)** Effects of BYQZF on the ΔΨm of PD cells after transfection with a parkin-knockdown plasmid. **(A)** The mitochondria of living cells were labeled with tetramethylrhodamine. **(B)** Effects of BYQZF on the relative fluorescence intensity of mitochondria in PD cells after with transfection with a parkin-knockdown plasmid. Data are expressed as the means ± standard deviations (*n* = 3). Compared with the negative control (NC) group, **p < 0.01; compared with the negative model (NM) group, ^#^ *p* < 0.05, ^##^ *p* < 0.01; compared with the negative treatment (NT) group, ^△^ *p* < 0.05, ^△△^ *p* < 0.01; compared with the parkin-knockdown control (PKC) group, ^▲^ *p* < 0.05, ^▲▲^ *p* < 0.01.

Parkin, which is a common genetic susceptibility gene in patients with PD, plays key roles in cell death induced by mitochondrial dysfunction (Bai et al., 2020). Therefore, we evaluated the effects of BYQZF on mitochondrial function in PD cells after transfection with a parkin-knockdown plasmid. After treatment with MPP^+^, the cell survival rate (Figure 4A), ATP level (Figure 4B), and relative fluorescence intensity of mitochondria (Figure 4C) were obviously decreased in each model group (*p* < 0.01). Additionally, the cell survival rate, ATP level, and relative fluorescence intensity of mitochondria decreased significantly (*p* < 0.01) in parkin-knockdown SH-SY5Y cells. Notably, treatment with BYQZF markedly enhanced these parameters in the negative treatment group (*p* < 0.01; *p* < 0.05). However, the cell survival rate, ATP level, and relative fluorescence intensity of mitochondria did not increase after treatment with BYQZF in the parkin-knockdown model group (*p* > 0.05), suggesting that the effects of BYQZF on mitochondrial function were significantly lower after transfection with the parkin-knockdown plasmid.

### Effects of Bu-Yin-Qian-Zheng Formula on Mitochondrial Morphology in Parkin-Knockdown Parkinson’s Disease Cells

**FIGURE 5:**
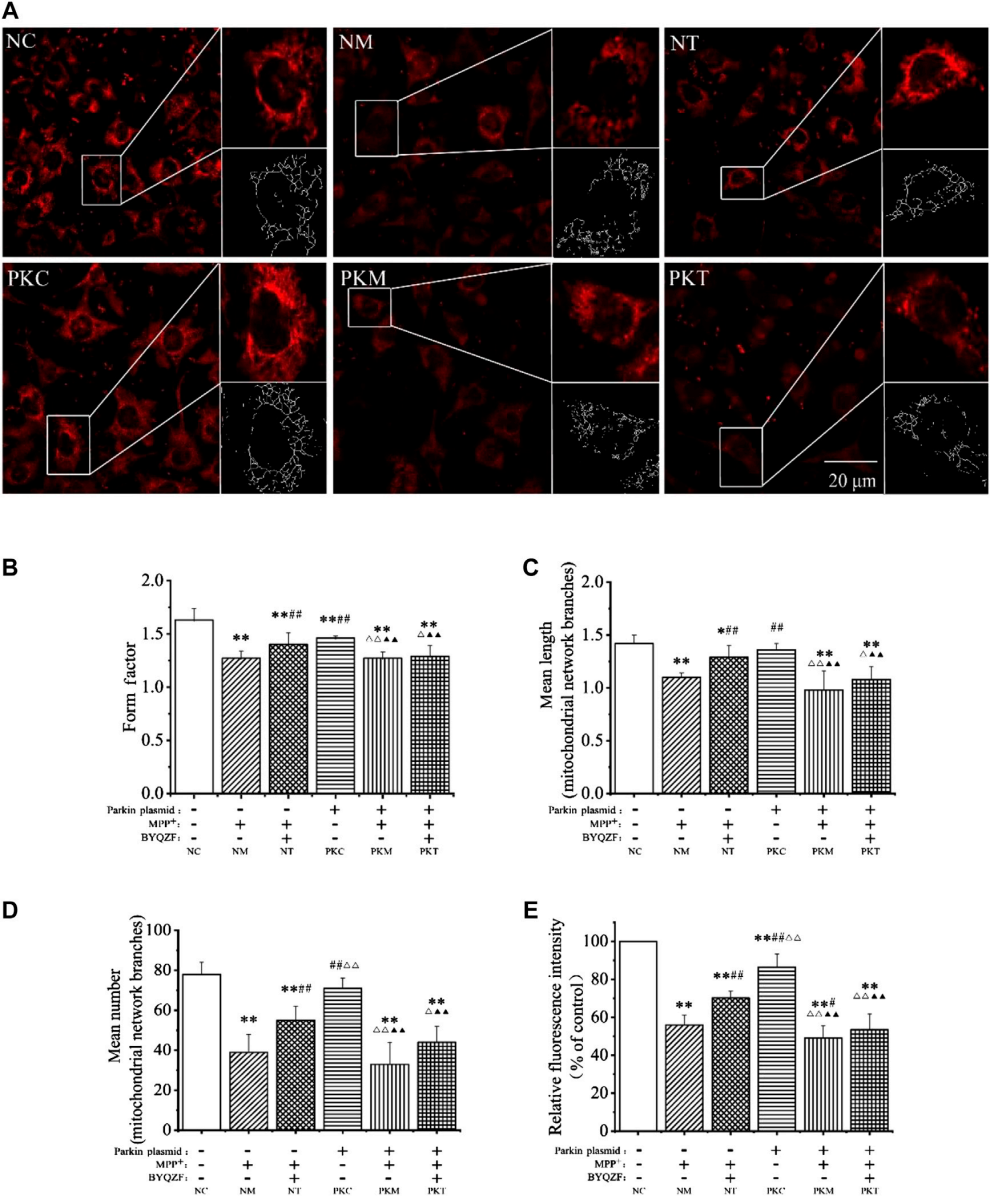
Effects of BYQZF on the mitochondrial morphology of PD cells after transfection with a parkin-knockdown plasmid. **(A)** Mitochondria were labeled with MTR and assessed by confocal fluorescence microscopy (100×). **(B)** Form factor of mitochondria. **(C)** Mean length of mitochondrial network branches. **(D)** Mean number of mitochondrial network branches. **(E)** Analysis of mitochondrial activity. Data are expressed as the means ± standard deviations (*n* = 3). Compared with the negative control (NC) group, ^*^ *p* < 0.05, **p < 0.01; compared with the negative model (NM) group, ^#^ *p* < 0.05, ^##^ *p* < 0.01; compared with the negative treatment (NT) group, ^△^ *p* < 0.05, ^△△^ *p* < 0.01; compared with the parkin-knockdown control (PKC) group, ^▲▲^ *p* < 0.01.

Mitochondrial morphology is highly variable in different biological environments. Mitochondria are fragmented and spherical and show a lack of dynamics and network formation when damaged. In this study, the probe MTR was used to detect mitochondrial morphology. As shown in Figure 5A, the cells were full of clear nuclei, and mitochondria were distributed around the nucleus with long protrusions in the negative control group. Compared with the negative control group, the number of cells was reduced and mitochondria were shorter and rounder in each model group and in the parkin-knockdown control group. BYQZF alleviated these effects in the negative treatment group. However, there were no significant changes in mitochondrial morphology between the parkin-knockdown treatment group and parkin-knockdown model group.

As shown in Figures 5B–E, the mitochondrial form factor, mean length and number of mitochondrial network branches, and mitochondrial activity were significantly reduced in each model group after treatment with MPP^+^ (*p* < 0.01). Moreover, the form factor and mitochondrial activity decreased significantly (*p* < 0.01) in parkin-knockdown SH-SY5Y cells; the mean length and number of mitochondrial network branches also decreased; however, these differences were not significant (*p* > 0.05). After treatment with BYQZF, the mitochondrial form factor, mean length and number of mitochondrial network branches, and mitochondrial activity were significantly increased in the negative treatment group (*p* < 0.01). Compared with the parkin-knockdown model group, there were no obvious differences in any of these parameters in the parkin-knockdown treatment group (*p* > 0.05).

### Effects of Bu-Yin-Qian-Zheng Formula on Expression of PINK1 and Parkin Protein in Parkin-Knockdown Parkinson’s Disease Cells

**FIGURE 6:**
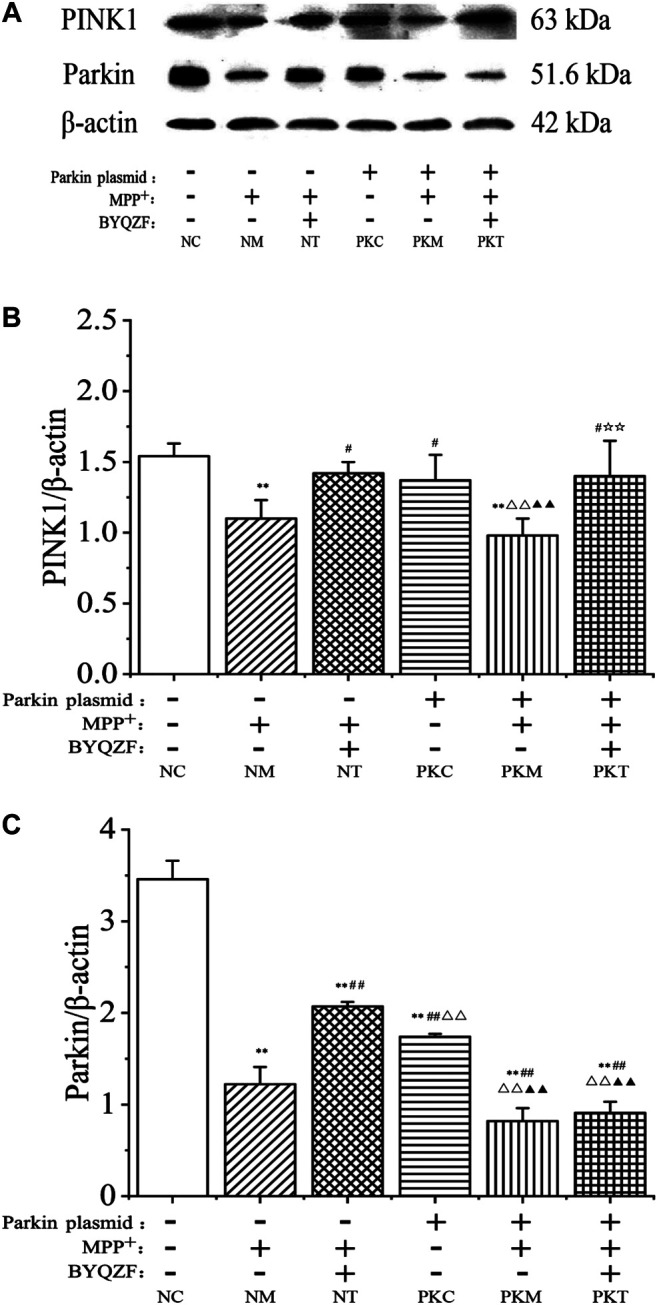
Effects of BYQZF on the expression of PINK1 and parkin proteins in PD cells after transfection with a parkin-knockdown plasmid. **(A)** Western blotting to detect PINK1 and parkin proteins expression. **(B)** Quantification of PINK1 protein levels. **(C)** Quantification of parkin protein levels. Data are expressed as the means ± standard deviations (*n* = 3). Compared with the negative control (NC) group, **p < 0.01; compared with the negative model (NM) group, ^##^ *p* < 0.01; compared with the negative treatment (NT) group, ^△△^ *p* < 0.01; compared with the parkin-knockdown control (PKC) group, ^▲▲^ *p* < 0.01.

As shown in Figure 6, the western blotting results showed that the expressions of parkin and PINK1 protein in each model group decreased noticeably after MPP^+^ treatment (*p* < 0.01). In addition, the expression of parkin protein was significantly reduced in parkin-knockdown SH-SY5Y cells (*p* < 0.01). BYQZF increased the expression of PINK1 and parkin protein in the negative treatment group (*p* < 0.01; Figures 6B,C). However, the expression of parkin protein did not increase after treatment with BYQZF in the parkin-knockdown model group (*p* > 0.05; Figure 6C).

### Effects of Bu-Yin-Qian-Zheng Formula on Expression of Mitochondrial Fusion and Fission Proteins in Parkin-Knockdown Parkinson’s Disease Cells

**FIGURE 7:**
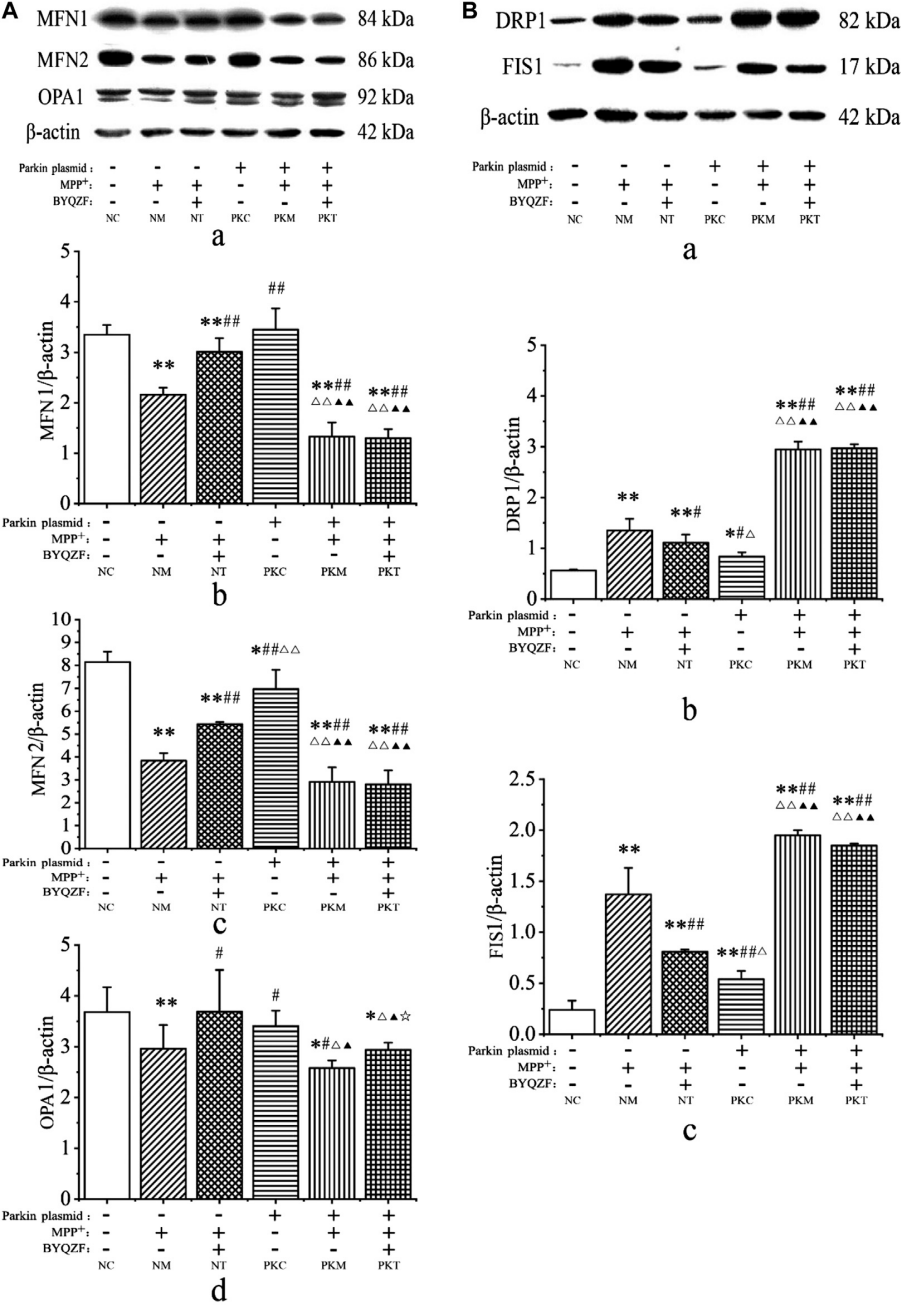
Analysis of expression of mitochondrial fusion and fission proteins. **(A)** Effects of BYQZF on the expression of mitochondrial fusion proteins after transfection with a parkin-knockdown plasmid. **(a)** Western blotting to detect MFN1, MFN2, and OPA1. Quantification of MFN1 **(b)**, MFN2 **(c)**, and OPA1 **(d)** levels. **(B)** Effects of BYQZF on expression of mitochondrial fission proteins after transfection with a parkin-knockdown plasmid. **(a)** Western blotting to detect DRP1 and FIS1. Quantification of DRP1 **(b)** and FIS1 **(c)** levels. Data are expressed as the means ± standard deviations (*n* = 3). Compared with the negative control (NC) group, ^*^ *p* < 0.05, **p < 0.01; compared with the negative model (NM) group, ^#^ *p* < 0.05, ^##^ *p* < 0.01; compared with the negative treatment (NT) group, ^△^ *p* < 0.05, ^△△^ *p* < 0.01; compared with the parkin-knockdown control (PKC) group, ^▲^ *p* < 0.05, ^▲▲^ *p* < 0.01; compared with the parkin-knockdown model (PKM) group, ^☆^ *p* < 0.05.

Mitochondrial dynamics are critical for regulating the mitochondrial morphology, number, and function (Meyer et al., 2017). Therefore, we next investigated the effects of BYQZF on mitochondrial dynamics in PD cells. Western blotting results showed that the expression levels of mitochondrial fusion proteins (MFN1, MFN2, and OPA1) were significantly reduced in each model group after MPP^+^ treatment (*p* < 0.01, *p* < 0.05; Figure 7A). The expression of MFN2 protein decreased in parkin-knockdown SH-SY5Y cells (*p* < 0.05). Additionally, BYQZF increased the expression of mitochondrial fusion proteins (MFN1, MFN2, and OPA1) in the negative treatment group (*p* < 0.01, *p* < 0.05) and expression of OPA1 protein in the parkin-knockdown model group (*p* < 0.05). However, the expression of mitochondrial fusion proteins (MFN1 and MFN2) did not increase after treatment with BYQZF in the parkin-knockdown model group (*p* > 0.05).

As shown in Figure 7B, MPP^+^ increased the expression of mitochondrial fission proteins (DRP1 and FIS1) in each model group (*p* < 0.01). After parkin knockdown, the expression of mitochondrial fission proteins (DRP1 and FIS1) increased significantly (*p* < 0.01, *p* < 0.05). Moreover, after BYQZF treatment, the expression of DRP1 and FIS1 was decreased in the negative treatment group (*p* < 0.01, *p* < 0.05). Compared with the parkin-knockdown model group, there were no significant changes in the expression levels of DRP1 and FIS1 in the parkin-knockdown treatment group (*p* > 0.05).

## Discussion

The protective effects of BYQZF on mitochondrial function have been confirmed in *in vitro* and *in vivo* studies (Zhang et al., 2013; Zhang et al., 2018; Gai et al., 2019); parkin was found to play an important role in this process. However, the mechanisms by which BYQZF ameliorates mitochondrial dysfunction via parkin are unclear. In the current study, BYQZF showed reduced protective effects on mitochondrial function when parkin was knocked down. This is the first study to demonstrate that BYQZF ameliorated mitochondrial dysfunction through parkin.

In this study, the SH-SY5Y cell line has been used as a model for PD since it has the machinery to synthesize DA (Krishna et al., 2014; Xicoy et al., 2017). MPP^+^ is one of the most frequently used neurotoxins in creating cellular models and studying critical aspects of PD in SH-SY5Y cells (Yang et al., 2018; Chanthammachat and Dharmasaroja, 2019). Therefore, MPP^+^-induced SH-SY5Y neurotoxicity has been broadly utilized to create cellular models and study the mechanisms of PD.

Mitochondria play key roles in energy modulation (Nunnari and Suomalainen, 2012). The maintenance of mitochondrial functionality is essential for neuronal survival in PD (Nunnari and Suomalainen, 2012; Schapira, 2012; Bankapalli et al., 2020) because MPP^+^ inhibits mitochondrial complex I and thus causes mitochondrial dysfunction (Bender et al., 2006; Rao et al., 2020). The electrochemical gradient (ΔΨm) across the inner mitochondrial membrane is important for ATP production (Finkel et al., 2015). These factors reflect the state of mitochondrial function. Moreover, several studies have shown that parkin plays a direct role in mitochondrial biogenesis (Sliter et al., 2018; Noda et al., 2020), and mutation or deletion of parkin can lead to changes in mitochondrial function and trigger PD-like symptoms (Dodson and Guo, 2007; Zhu et al., 2018). For example, Stevens et al. (2015) found that loss of parkin may damage mitochondrial health by impairing mitochondrial biogenesis and eventually lead to cell death. Additionally, Lim et al. (2012) found that impaired mitochondria may contribute to DA neurodegeneration in parkin-related cases. In this study, a parkin-knockdown cell model was successfully constructed, and the ΔΨm and ATP levels were lower in the parkin-knockdown control group. Taken together, these data suggest that parkin maintains the normal state of the mitochondria and can be used as a mitochondrion-targeted protein for applications in PD therapy (Plotegher and Duchen, 2017).

BYQZF is composed of Amur cork tree bark (Huangbai), prepared Rehmannia root (Shudihuang), common anemarrhena (Zhimu), tortoise plastron (Guiban), Chinese scorpion (detoxicated; Quanxie), stiff silkworm (Jiangcan), and giant typhonium tuber (Baifuzi). Recent studies have shown that some active ingredients in BYQZF can protect the activity of substantia nigra DA neurons. For example, catalpol, a component of shú dì huáng, alleviates MPTP-induced degeneration of DA neurons *in vivo* (Wang et al., 2019). Additionally, mangiferin, a chemical constituent of zhî mu, significantly upregulates substantia nigra dopamine concentrations and decreases the effects of neurotoxins, thereby affecting mitochondrial function in PD (Kavitha et al., 2013; Feng et al., 2019). Extracts from guî bãn promote the differentiation of neural stem cells into dopaminergic neurons by regulating *OTX2* expression (Zhou et al., 2017). Notably, we previously found that BYQZF ameliorated mitochondrial dysfunction and promoted the expression of parkin protein (Gai et al., 2019). However, further studies are required to determine whether parkin is a mitochondrial target for BYQZF for treating PD.

We found that BYQZF rescued MPP^+^-induced mitochondrial alterations, including ΔΨm and ATP levels in the negative treatment group, consistent with a previous study (Gai et al., 2019). However, BYQZF did not rescue mitochondrial dysfunction in MPP^+^-induced SH-SY5Y cells transfected with the parkin-knockdown plasmid. Accordingly, parkin may be a mitochondrial target for BYQZF and can protect mitochondrial function in the treatment of PD. However, the exact mechanisms through which BYQZF affects mitochondrial functions are poorly understood, and identification of these mechanisms may facilitate the development of novel treatments of PD. Thus, further studies are required.

Mitochondrial morphology, which can be regulated by the balance between fusion and fission (Li et al., 2016), is intimately linked with mitochondrial function (Nunnari and Suomalainen, 2012) and facilitates specific cellular activities (Lisowski et al., 2018). Notably, parkin can maintain mitochondrial morphology and function by regulating the state of fission and fusion. Additionally, most studies in mammalian cells have shown that parkin acts in the direction of fusion and that parkin overexpression leads to the growth of interconnected mitochondrial networks (Tang et al., 2016). Outer mitochondrial membrane fusion is mediated by MFNs (MFN1 and MFN2), whereas inner membrane fusion is mediated by OPA1 (Wallace, 2012; Pernas and Scorrano, 2016). Mitochondrial fission is mediated by DRP1 and FIS1 (Schapira, 2012; Pernas and Scorrano, 2016). In this study, we found that the mitochondrial form factor, mean length and number of mitochondrial network branches, mitochondrial activity, and expression of MFN2 protein were decreased, whereas DRP1 and FIS1 expression levels were increased in the parkin-knockdown control group. These results are consistent with those of previous studies demonstrating that parkin downregulation exacerbates mitochondrial fragment via regulation of mitochondrial fission and fusion (Pinto and Nissanka, 2018; Noda et al., 2020). Overall, these findings highlight the importance of parkin not only as a genetic marker of PD but also as an important factor in its pathogenesis, via a mechanism related to the regulation of mitochondrial dynamics. The precise mechanisms through which BYQZF affects mitochondrial functions via parkin may related to the regulation of mitochondrial dynamics.

Parkin is involved in the balance between fusion and fission, functioning to maintain mitochondrial morphology. MFN1, MFN2, DRP1, and FIS1 are the main substrates of parkin (Kandimalla and Reddy, 2016). In this study, we found that parkin protein expression was downregulated in MPP^+^-induced SH-SY5Y cells; however, treatment with BYQZF blocked this effect, promoting parkin protein expression after transfection of cells with the negative plasmid. In contrast, BYQZF did not enhance parkin expression following transfection with a parkin-knockdown plasmid. Moreover, changes in mitochondrial morphology were consistent with parkin protein expression. Additionally, we found that changes in the expression of MFN1 and MFN2 were consistent with those in the expression of parkin protein, whereas changes in DRP1 and FIS1 expression were opposite those of parkin protein. These findings suggest that knockdown of parkin suppressed the regulatory effects of BYQZF on mitochondrial fusion and fission proteins. Accordingly, parkin may mediate the effects of BYQZF on mitochondrial function and biogenesis by regulating fusion and fission processes. In this study, we evaluated the potential mechanisms by which BYQZF ameliorated mitochondrial dysfunction by regulating mitochondrial dynamics via parkin in PD. However, some hypotheses regarding the mechanism by which parkin mutations or loss of function lead to PD have suggested that parkin deficiency is associated with mitochondrial autophagy. We have not yet investigated the effect of BYQZF on mitochondrial autophagy. Whether the mechanism through which BYQZF affects mitochondrial functions via parkin is related to the regulation of mitochondrial autophagy? This is exactly what we need to study further.

## Conclusion

In this study, we assessed the mechanisms through which parkin and BYQZF affect mitochondrial function. Our results showed that the effects of BYQZF on mitochondrial dysfunction were dependent on parkin expression. Thus, we identified parkin as a mitochondrial target for the treatment with BYQZF in PD. Additionally, we found that the mechanism through which BYQZF affected mitochondrial function involved regulation of mitochondrial dynamics via parkin. Taken together, our findings demonstrate that BYQZF ameliorated mitochondrial dysfunction by regulating mitochondrial dynamics via parkin, suggesting that this traditional Chinese medicine can be applied for treating PD.

## Data Availability Statement

The original contributions presented in the study are included in the article/Supplementary Material, further inquiries can be directed to the corresponding authors.

## Author Contributions

H-JM and CG contributed to the interpretation of the results and writing of the manuscript. H-MS contributed to the study design and interpretation of the results. Y-SG and Z-YG instructed experiments. YC, W-DF, C-CC, J-KZ, Y-XZ, L-PY, and Z-YG conducted the experiments. All authors reviewed the manuscript.

## Funding

This work is supported by the National Natural Science Foundation of China (Grant Nos. 81774110 and 81573773).

## Conflict of Interest

The authors declare that the research was conducted in the absence of any commercial or financial relationships that could be construed as a potential conflict of interest.
