# Supplementary figures and images for: Bu-Yin-Qian-Zheng Formula Ameliorates MPP+-Induced Mitochondrial Dysfunction in Parkinson’s Disease via Parkin
Source: Front Pharmacol. 2020 Dec 18;11:577017. doi: 10.3389/fphar.2020.577017 (PMC7793772; doi:10.3389/fphar.2020.577017)

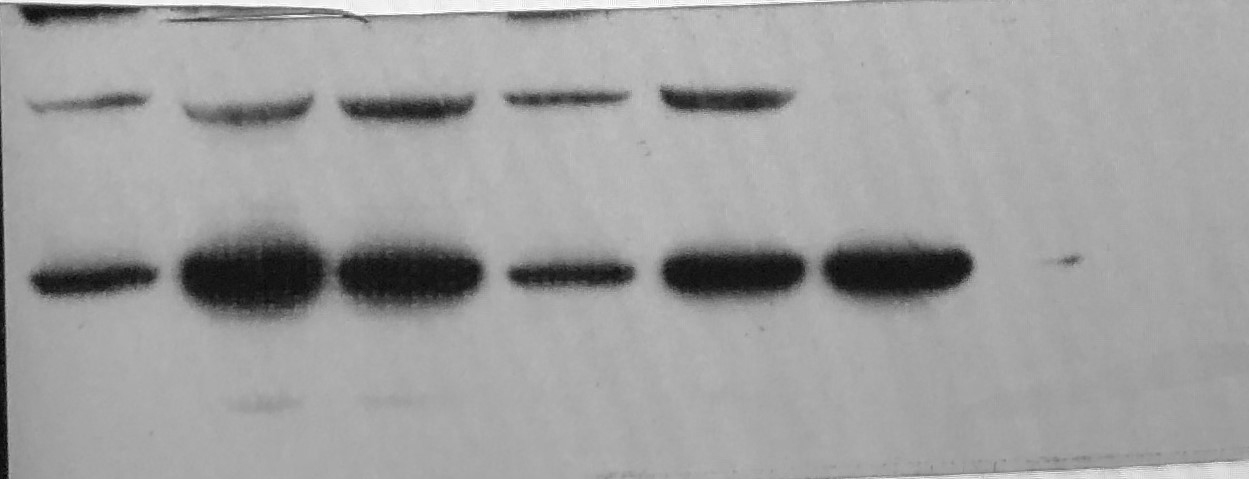

Supplement: Supplementary file 1 [file image1.jpeg]

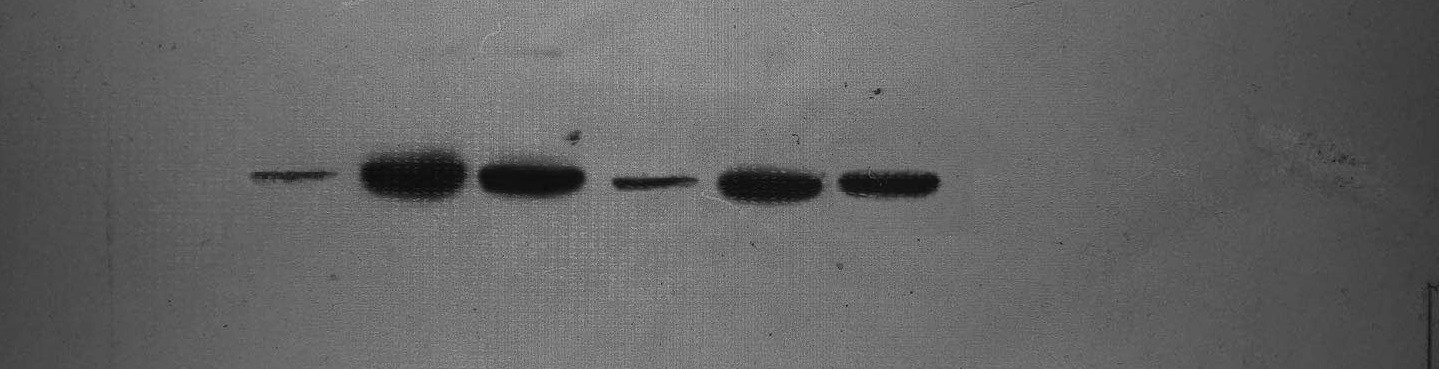

Supplement: Supplementary file 2 [file image2.jpeg]

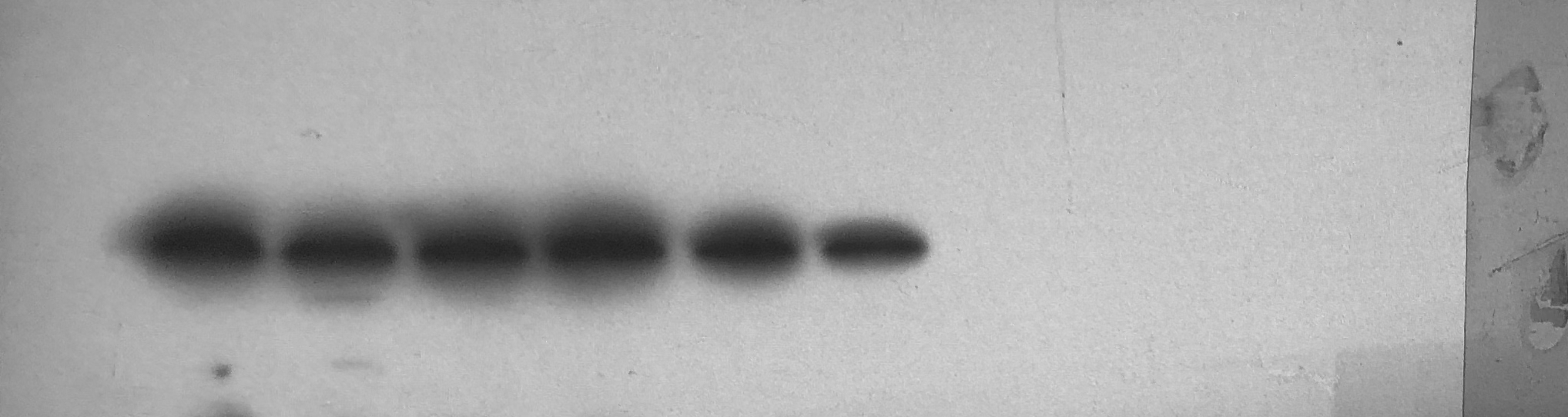

Supplement: Supplementary file 3 [file image3.tif]

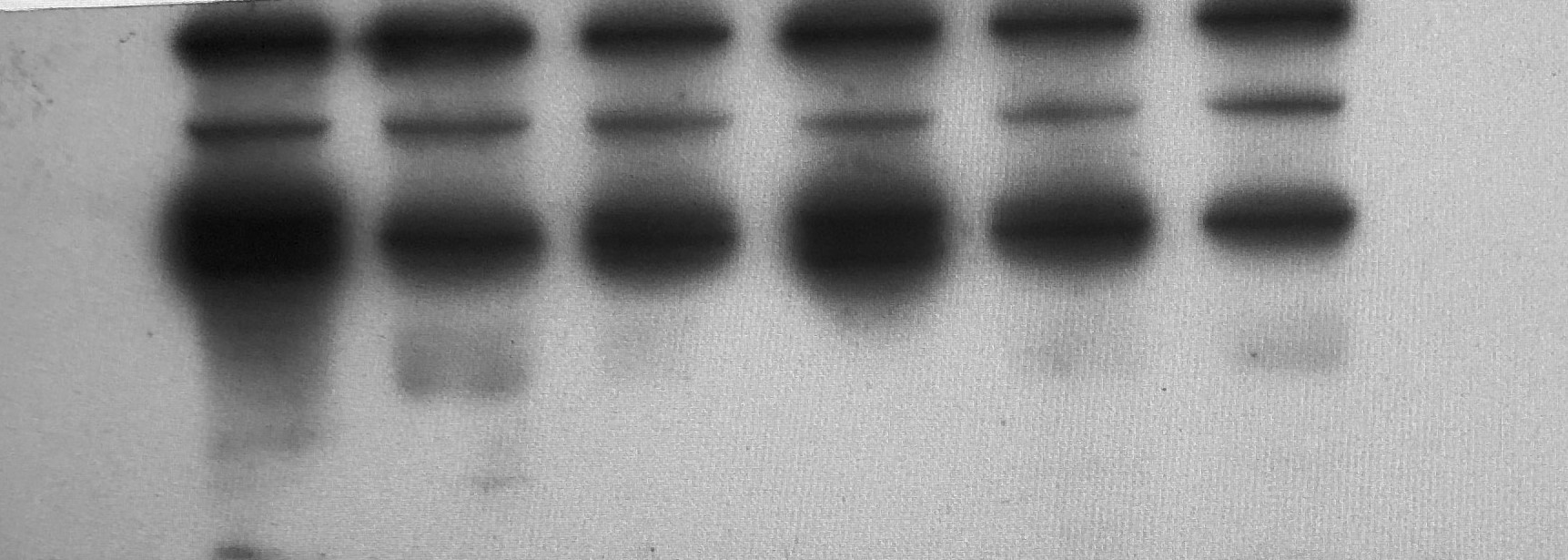

Supplement: Supplementary file 4 [file image4.jpeg]

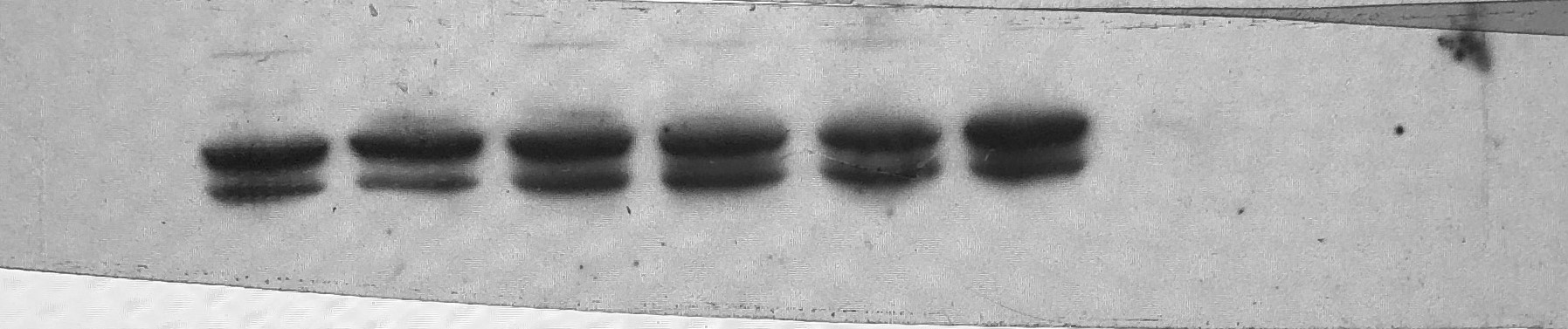

Supplement: Supplementary file 5 [file image5.jpeg]

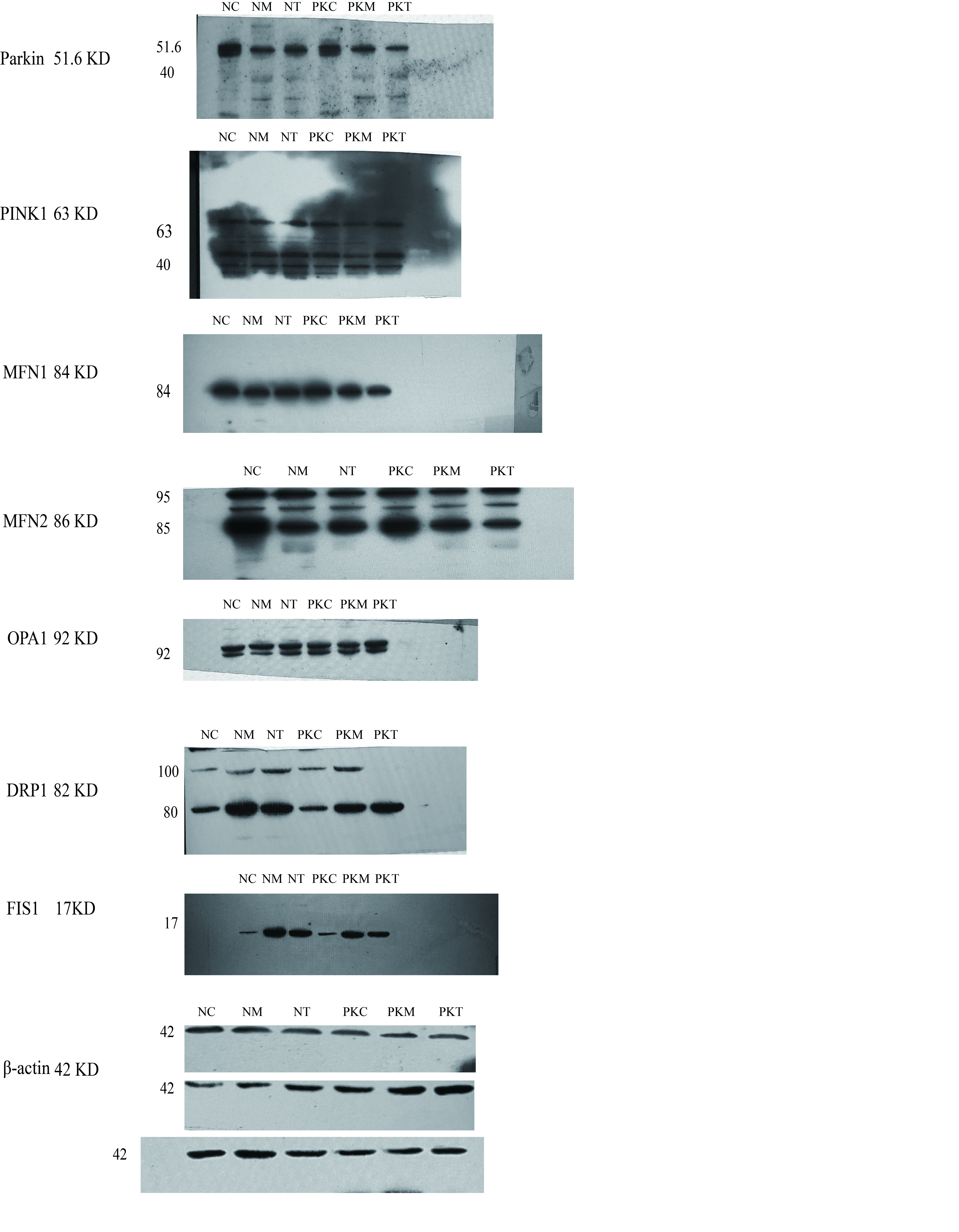

Supplement: Supplementary file 6 [file image6.jpeg]

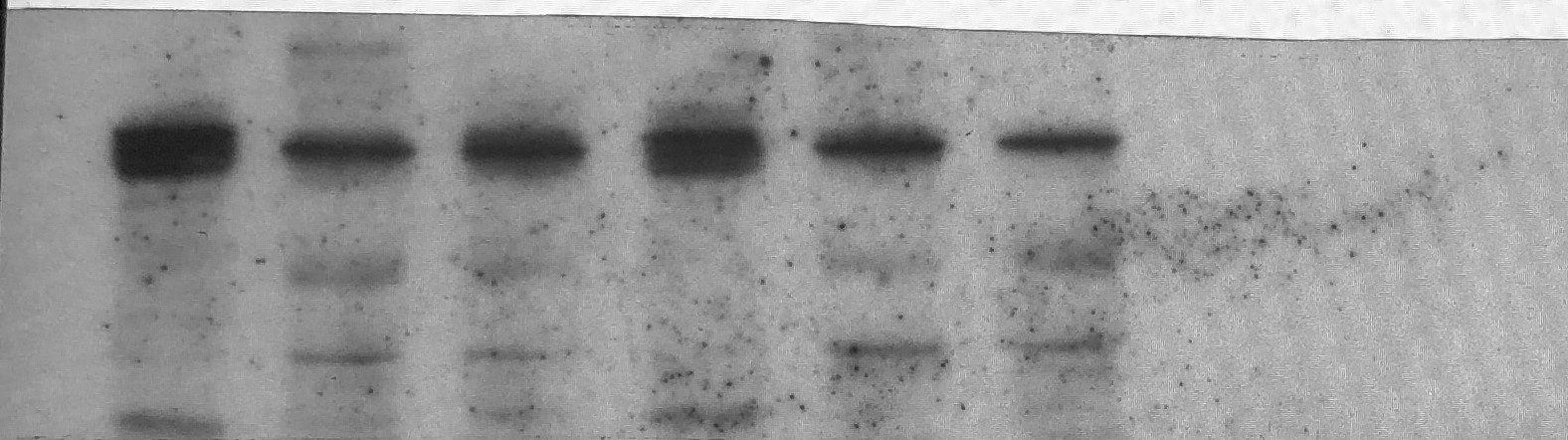

Supplement: Supplementary file 7 [file image7.jpeg]

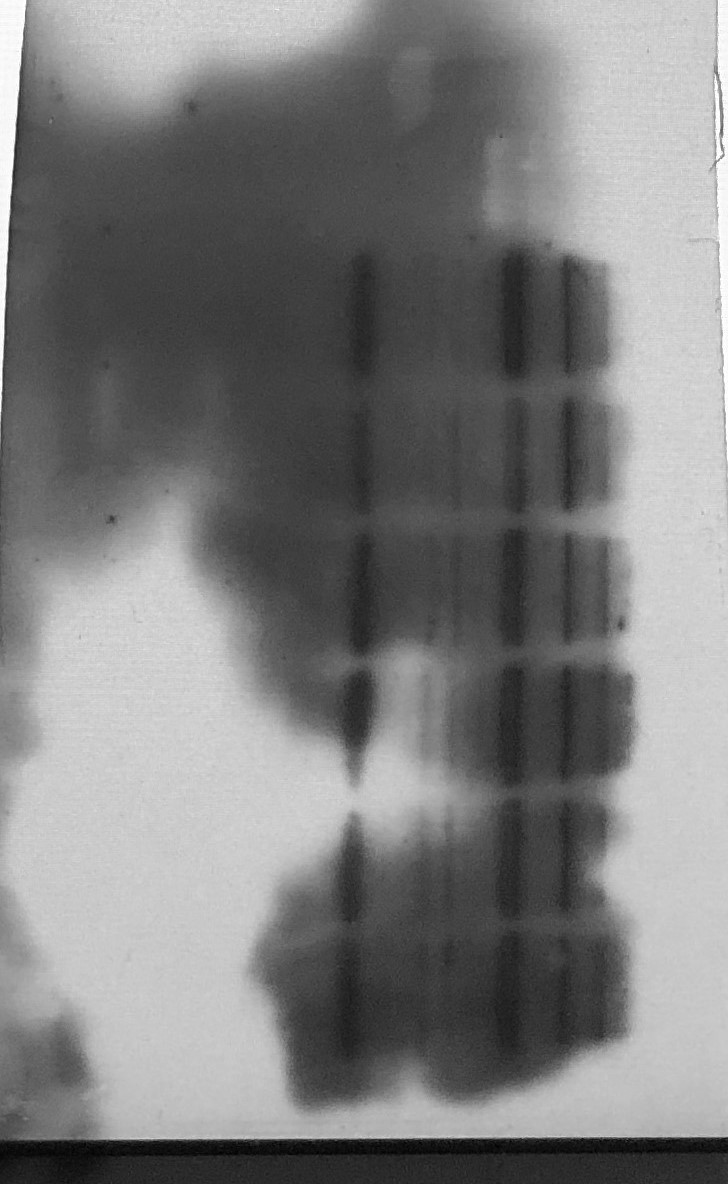

Supplement: Supplementary file 8 [file image8.jpeg]

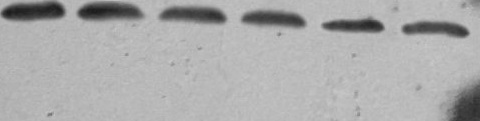

Supplement: Supplementary file 9 [file image9.jpeg]

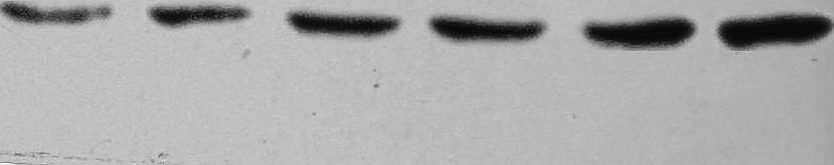

Supplement: Supplementary file 10 [file image10.jpeg]

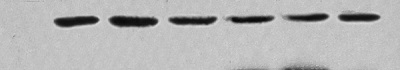

Supplement: Supplementary file 11 [file image11.jpeg]
